# Nirmatrelvir-ritonavir use among adults hospitalized with COVID-19 during the Omicron phase of the COVID-19 pandemic, Canadian Nosocomial Infection Surveillance Program

**DOI:** 10.14745/ccdr.v49i78a07

**Published:** 2023-08-01

**Authors:** Robyn Mitchell, Diane Lee, Linda Pelude, Jeannette Comeau, John Conly, Chelsey Ellis, Jennifer Ellison, John Embil, Gerald Evans, Lynn Johnston, Jennie Johnstone, Kevin Katz, Pamela Kibsey, Bonita Lee, Marie-Astrid Lefebvre, Yves Longtin, Allison McGeer, Dominik Mertz, Jessica Minion, Stephanie Smith, Jocelyn Srigley, Kathryn Suh, Jen Tomlinson, Alice Wong, Nisha Thampi, Charles Frenette

**Affiliations:** 1 Public Health Agency of Canada, Ottawa, ON; 2 IWK Health Centre, Halifax, NS; 3 University of Calgary, Calgary, AB; 4 The Moncton Hospital, Moncton, NB; 5 Alberta Health Services, Calgary, AB; 6 Health Sciences Centre, Winnipeg, MB; 7 Queen’s University, Kingston, ON; 8 QEII Health Sciences Centre, Halifax, NS; 9 Sinai Health, Toronto, ON; 10 North York General Hospital, Toronto, ON; 11 Royal Jubilee Hospital, Victoria, BC; 12 Stollery Children’s Hospital, Edmonton, AB; 13 McGill University Health Centre, Montréal, QC; 14 SMBD-Jewish General Hospital, Montréal, QC; 15 McMaster University and Hamilton Health Sciences, Hamilton, ON; 16 Saskatchewan Health Authority, Regina, SK; 17 University of Alberta Hospital, Edmonton, AB; 18 BC Women’s and BC Children’s Hospital, Vancouver, BC; 19 The Ottawa Hospital, Ottawa, ON; 20 Royal University Hospital, Saskatoon, SK; 21 Children’s Hospital of Eastern Ontario, Ottawa, ON

**Keywords:** nirmatrelvir-ritonavir, COVID-19, hospitalized patients, Omicron

## Abstract

**Background:**

Recent studies have demonstrated the effectiveness of nirmatrelvir-ritonavir in reducing the risk of progression to severe disease among outpatients with mild to moderate coronavirus disease 2019 (COVID-19); however, data are limited regarding the use and role of nirmatrelvir-ritonavir among hospitalized patients. This study describes the use and outcomes of nirmatrelvir-ritonavir among adults hospitalized with COVID-19 in a sentinel network of Canadian acute care hospitals during the Omicron variant phase of the pandemic.

**Methods:**

The Canadian Nosocomial Infection Surveillance Program conducts surveillance of hospitalized patients with COVID-19 in acute care hospitals across Canada. Demographic, clinical, treatment and 30-day outcome data were collected by chart review by trained infection control professionals using standardized questionnaires.

**Results:**

From January 1 to December 31, 2022, 13% (n=490/3,731) of adult patients (18 years of age and older) hospitalized with COVID-19 in 40 acute care hospitals received nirmatrelvir-ritonavir either at admission or during hospitalization. Most inpatients who received nirmatrelvir-ritonavir, 79% of whom were fully vaccinated, had at least one pre-existing comorbidity (97%) and were of advanced age (median=79 years). Few were admitted to an intensive care unit (2.3%) and among the 490 nirmatrelvir-ritonavir treated inpatients, there were 13 (2.7%) deaths attributable to COVID-19.

**Conclusion:**

These findings from a large sentinel network of Canadian acute-care hospitals suggest that nirmatrelvir-ritonavir is being used to treat adult COVID-19 patients at admission who are at risk of progression to severe disease or those who acquired COVID-19 in hospital. Additional research on the efficacy and indications for nirmatrelvir-ritonavir use in hospitalized patients is warranted to inform future policies and guidelines.

## Introduction

Vaccination against severe acute respiratory syndrome coronavirus 2 (SARS-CoV-2) remains the most effective intervention to prevent severe coronavirus disease 2019 (COVID-19) related illness and death ((1–6)). For those who become infected, antiviral therapies such as nirmatrelvir-ritonavir are valuable tools to improve patient outcomes and reduce the burden on healthcare systems. A recent randomized controlled trial demonstrated that treatment with nirmatrelvir-ritonavir among unvaccinated, non-hospitalized adults during the pre-Delta and Delta pandemic phases resulted in an 89% reduction in hospitalization or death ((7)). Recent observational studies have shown the benefit of nirmatrelvir-ritonavir in reducing the risk of hospitalization and death among outpatients with mild or moderate COVID-19 who are at risk for progression to severe disease ((8–11)).

Nirmatrelvir-ritonavir was approved for use by Health Canada on January 17, 2022, for treating adults with mild to moderate COVID-19 infection who are at high risk for progression to severe disease, including hospitalization and death ((12)). A recent observational study from Ontario, Canada, found that outpatient use of nirmatrelvir-ritonavir during an Omicron-dominant period between April and August 2022 was associated with a significant reduction in the odds of hospital admission from COVID-19 or all-cause mortality. The largest benefits were observed among those who were under-vaccinated or unvaccinated and those 70 years of age or older ((13)). Information regarding the use of nirmatrelvir-ritonavir among hospitalized patients with mild to moderate disease during the Omicron phase of the pandemic is limited. To help inform future policies and guidelines, we sought to describe the use and outcomes of nirmatrelvir-ritonavir among adult patients hospitalized with COVID-19 in a sentinel network of Canadian acute care hospitals.

## Methods

The Canadian Nosocomial Infection Surveillance Program (CNISP) is a collaboration between the Public Health Agency of Canada, the Association of Medical Microbiology and Infectious Disease Canada and sentinel hospitals across Canada ((14)). The CNISP conducts surveillance of healthcare-associated (HA) infections among hospitalized adult and paediatric patients, including HA viral respiratory infections. In March 2020, surveillance was expanded to include patients of all ages hospitalized with COVID-19, in addition to patients with HA viral respiratory infection. Beginning on January 1, 2022, eligibility for the inclusion of patients with COVID-19 was restricted to those who were admitted due to COVID-19 or acquired COVID-19 while in hospital.

Demographic, clinical, treatment and 30-day outcome data were collected by trained infection control professionals by chart review and submitted to the Public Health Agency of Canada through a secure online platform, the Canadian Network for Public Health Intelligence, using a standardized protocol and data collection form. Information on initiation of nirmatrelvir-ritonavir was collected between January 1 and December 31, 2022. Data on initiation of nirmatrelvir-ritonavir prior to admission were not systematically recorded in the patient chart; therefore, patients who started nirmatrelvir-ritonavir prior to admission were excluded from the analysis. Outcomes were identified at 30 days from the date of the first positive reverse transcription-polymerase chain reaction test. Attributable mortality was defined as COVID-19 being the cause of death or contributing to death. A HA case was defined as a patient 1) with symptom onset or positive test seven or more calendar days after admission to hospital, or 2) who was readmitted with a positive test within less than seven days after discharge from hospital, or 3) who was most likely a HA case based on best clinical judgment (e.g. symptom onset prior to the seventh day but known epidemiological link to a positive inpatient or staff case).

The primary analysis describes adult patients, 18 years of age and older, who received nirmatrelvir-ritonavir at admission or during hospitalization. A subgroup analysis was conducted among HA COVID-19 adult patients to compare treated to non-treated patients. Paediatric patients, younger than 18 years, were excluded from the analysis. Chi-squared test or Fisher’s exact test were used to compare proportions and the Kruskal-Wallis rank sum test was used to compare medians. Missing and incomplete data for individual variables were excluded from analyses, therefore denominators may vary. Provinces were grouped into three regions: Western (British Columbia, Alberta, Saskatchewan and Manitoba); Central (Ontario and Québec); and Eastern (Nova Scotia, New Brunswick, Prince Edward Island and Newfoundland and Labrador). Analyses were conducted using R version 4.0.5.

## Results

From January 1, 2022, to December 31, 2022, 40 CNISP-participating hospitals in nine provinces submitted data on 3,731 adult inpatients with laboratory-confirmed COVID-19 for whom information on receipt of nirmatrelvir-ritonavir was available. During this period, 13% (n=490/3,731) were prescribed nirmatrelvir-ritonavir either at admission or during hospitalization. Among all inpatients hospitalized with COVID-19, the proportion who received nirmatrelvir-ritonavir either at admission or during hospitalization was significantly higher in Eastern Canada (28%), followed by Central (18%) and Western Canada (3%) (*p*<0.001) (**Table 1**).

**Table 1:** Summary of participating hospitals that provided detailed patient information, January 1–December 31, 2022

Region	Reporting hospitals (n=40)	Adults who received nirmatrelvir-ritonavir among patients hospitalized with COVID-19 (n=3,731)	
		n	%
Western Canada^a^	15	43/1,370	3.1%
Central Canada^b^	19	397/2,180	18.2%
Eastern Canada^c^	6	50/181	27.6%

Abbreviation: COVID-19, coronavirus disease 2019

^a^ Western refers to British Columbia, Alberta, Saskatchewan and Manitoba

^b^ Central refers to Ontario and Québec

^c^ Eastern refers to Nova Scotia, New Brunswick, Prince Edward Island, and Newfoundland and Labrador

The median age of treated patients was 79 years (IQR: 68–87) and 53% (n=261/488) were male. Among those who were treated, nearly all (97%, n=469/486) had at least one pre-existing comorbidity. Hypertension (56%, n=273/486), chronic heart disease, excluding hypertension (37%, n=180/486) and diabetes (33%, 160/486) were the most reported conditions. Most treated inpatients (84%, n=388/463) were symptomatic and the most frequently reported symptoms were cough (49%, n=227/463), fever (30%, n=137/463) and weakness (29%, n=135/463). Of those who were asymptomatic, the majority (89%, n=67/75) had a HA COVID-19 infection. The median time from symptom onset to initiation of nirmatrelvir-ritonavir was two days (IQR: 1–4).

The median time from the date of a positive test to initiation of nirmatrelvir-ritonavir was one day (IQR: 0–1). Nearly half of treated patients (49%, n=226/464) acquired COVID-19 while in hospital. Five percent of inpatients (n=25/489) who received nirmatrelvir-ritonavir were admitted from a long-term care facility; of those, 64% (n=16/25) received nirmatrelvir-ritonavir within one day of admission.

The majority of treated patients (79%, n=282/356) had received two or more doses of a COVID-19 vaccine, while 6% (n=20/356) had received only one dose and 15% (n=54/356) were unvaccinated. The median time from the date of last COVID-19 vaccination to initiation of nirmatrelvir-ritonavir was 183 days (IQR: 120–304). The most common additional treatments among patients who received nirmatrelvir-ritonavir were corticosteroids (21%, n=101/487) and remdesevir (12%, n=60/484). Among inpatients with COVID-19 who received nirmatrelvir-ritonavir, 2.3% (n=11/481) were admitted to an intensive care unit (ICU), 1.1% (n=5/461) received mechanical ventilation and 6.1% (n=30/490) died (all-cause 30-day in-hospital death) (**Table 2**). Thirteen deaths (2.7%) among the 490 inpatients treated with nirmatrelvir-ritonavir were attributable to COVID-19; COVID-19 contributed to the death of seven patients and COVID-19 was the cause of death for six patients (1.2%) (Table 2).

**Table 2:** Frequency of 30-day outcomes among adults hospitalized with COVID-19 who received nirmatrelvir-ritonavir, January 1–December 31, 2022

30-day outcome	Adults hospitalized with COVID-19 who received nirmatrelvir-ritonavir (n=490)	
	n	%
ICU admission	11/481	2.3%
Mechanical ventilation	5/461	1.1%
Pulmonary embolism	4/483	0.8%
CPAP/BiPAP	2/460	0.4%
Dialysis initiated for COVID-19 complications	1/488	0.2%
Stroke	0/486	0%
Extracorporeal membrane oxygenation	0/459	0%
Patient died (all cause)	30/490	6.1%
**Death attributed to COVID-19**	**13/490**	**2.7%**
COVID-19 contributed to death	7/490	1.4%
COVID-19 was the cause of death	6/490	1.2%

Abbreviations: COVID-19, coronavirus disease 2019; CPAP/BiPAP, continuous positive airway pressure/bilevel positive airway pressure; ICU, intensive care unit

A subgroup analysis among HA patients found that the characteristics (e.g. age, sex, at least one pre-existing comorbidity and vaccination status) of untreated HA patients were similar to those of treated HA patients; however, ICU admissions were higher among untreated HA patients (8.3%, n=63/755) compared to treated HA patients (2.2%, n=5/223, *p*=0.002). Similarly, all-cause 30-day mortality was also higher among untreated HA patients (16%, n=18/226) compared to treated HA patients (8.0%, n=122/774, *p*=0.003) (**Table 3**).

**Table 3:** Patient characteristics and outcomes in adult inpatients with healthcare-associated COVID-19 by receipt of nirmatrelvir-ritonavir, January 1–December 31, 2022

Patient characteristics	Inpatients who received nirmatrelvir-ritonavir (n=226)		Inpatients who did NOT receive nirmatrelvir-ritonavir (n=774)		*p-*value
	n	%	n	%	
**Region**						
Western Canada^a^	8/226	3.5%	201/774	26.0%	<0.001
Central Canada^b^	188/226	83.2%	535/774	69.1%	<0.001
Eastern Canada^c^	30/226	13.2%	38/774	4.9%	<0.001
**Demographics**						
Median age (years)	77	68, 86	76	67, 85	0.62
Male sex	113/226	50.0%	423/770	54.9%	0.19
At least one pre-existing comorbidity	218/224	97.3%	745/764	97.5%	0.87
**Vaccination status**						
Unvaccinated	22/170	12.9%	65/659	9.9%	0.24
1 dose	9/170	5.3%	24/659	3.6%	0.33
2 or more doses	139/170	81.8%	570/659	86.5%	0.12
**Treatment**						
Anticoagulant	45/223	20.2%	338/756	44.7%	<0.001
Corticosteroid	25/225	11.1%	467/770	60.6%	<0.001
Remdesivir	22/223	9.9%	763/774	98.6%	<0.001
**30-day outcomes**						
ICU admission	5/223	2.2%	63/755	8.3%	0.002
Mechanical ventilation	2/224	0.9%	32/766	4.2%	0.018
Pulmonary embolism	0/223	0.0%	14/754	1.9%	0.049
CPAP/BiPAP	0/223	0.0%	29/756	3.8%	0.003
Dialysis initiated for COVID-19 complications	0/226	0.0%	5/767	0.7%	0.59
Stroke	0/225	0.0%	5/760	0.7%	0.59
Extracorporeal membrane oxygenation	0/223	0.0%	2/764	0.3%	>0.99
Patient died (all cause)	18/226	8.0%	122/774	15.8%	0.003
Death attributed to COVID-19	7/226	3.1%	68/774	8.8%	0.03

Abbreviations: COVID-19, coronavirus disease 2019; CPAP/BiPAP, continuous positive airway pressure/bilevel positive airway pressure; ICU, intensive care unit

^a^ Western refers to British Columbia, Alberta, Saskatchewan and Manitoba

^b^ Central refers to Ontario and Québec

^c^ Eastern refers to Nova Scotia, New Brunswick, Prince Edward Island, and Newfoundland and Labrador

## Discussion

Findings from a sentinel network of Canadian acute care hospitals found that, during the Omicron phase of the pandemic, 13% of adults hospitalized with COVID-19 received nirmatrelvir-ritonavir either at admission or during hospitalization. Nearly all inpatients, of whom 79% were fully vaccinated, had at least one pre-existing comorbidity and were of advanced age, which put them at increased risk of progression to severe disease or death. The proportion of severe outcomes (e.g. ICU admission and death attributable to COVID-19) at 30 days was low. Significant regional variation was observed in the use of nirmatrelvir-ritonavir, which is most likely related to differences in provincial policies and/or prescriber patterns, and possibly regional drug availability. However, it is difficult to attribute regional treatment differences to regional differences in patient populations, suggesting the need for more data on treatment indications for inpatients from which national treatment guidelines can be developed.

Few studies have evaluated the effectiveness of nirmatrelvir-ritonavir among hospitalized patients. However, a cohort study in Hong Kong during the Omicron pandemic phase demonstrated that initiation of nirmatrelvir-ritonavir treatment within five days of symptom onset among hospitalized patients 60 years of age and older or younger patients with at least one chronic disease was associated with a lower risk of in-hospital death compared to controls ((15)). A Chinese study suggested the potential role of early nirmatrelvir-ritonavir treatment for high-risk patients who are immunocompromised, including those who are hospitalized, to facilitate viral eradication ((16)). A retrospective cohort study of hospitalized patients with COVID-19 who did not initially require supplemental oxygen found that early initiation of nirmatrelvir-ritonavir was associated with significant reductions in risk of all-cause mortality and disease progression ((17)).

Our subgroup analysis found that HA COVID-19 adult inpatients who received nirmatrelvir-ritonavir were less frequently admitted to an ICU or less frequently died (30-day all-cause mortality) compared to non-treated HA COVID-19 adult inpatients. These results should be interpreted with caution as eligibility to receive nirmatrelvir-ritonavir was not determined (e.g. data on contraindications for nirmatrelvir-ritonavir were not collected) and due to the small sample size, a multivariable analysis was not conducted. Nonetheless, these preliminary results warrant further study. In addition to the treatment benefits among hospitalized patients reported in other recent studies, our findings suggest a role for nirmatrelvir-ritonavir treatment for adult patients with mild to moderate symptoms who are hospitalized for reasons unrelated to COVID-19 or who acquired COVID-19 in-hospital who are at high risk for progression to severe disease.

## Limitations

Our study has several limitations. This report describes early findings of the epidemiology of COVID-19 among inpatients who received nirmatrelvir-ritonavir in a subset of Canadian acute care hospitals; these findings may change as additional data become available. These analyses were descriptive in nature, and we cannot draw any causal inferences. Specifically, our findings should be interpreted with caution as there is potential for selection bias, given that our surveillance methodology did not identify eligibility of patients to receive treatment. Due to the regional variation in data submission and of nirmatrelvir-ritonavir use, our results may not be generalizable to all adult patients hospitalized in Canada. In addition, our cohort was limited to those with a positive test result for SARS-CoV-2 by polymerase chain reaction test and did not include inpatients with positive test result by only rapid antigen test, which may also limit the generalizability of our findings. Finally, we did not collect data on indications or drug contraindications to nirmatrelvir-ritonavir.

## Conclusion

Among adult patients hospitalized with COVID-19, we found that 13% received nirmatrelvir-ritonavir. Further study to monitor the use and effectiveness of nirmatrelvir-ritonavir among COVID-19 inpatients and other high-risk populations (e.g. long-term care residents) is critical to inform future policies and guidelines.
